# 

**Published:** 1852-07

**Authors:** 


### WESTERN JOURNAL

OF

### MEDICINE AND SURGERY:

EDITED BY

LUNSFORD P. YANDELL, M. D.,

PROFESSOR OF PHYSIOLOGY AND PATHOLOGICAL ANATOMY IN THE UNIVERSITY OF LOUISVILLE,

AND

THEODORE S. BELL, M. D.

CXLXI.—JULY, 1852.

THIRD SERIES.

Vol. X......No. I.

LOUISVILLE, KY:

PUBLISHED MONTHLY BY PRENTICE & HENDERSON,

PROPRIETORS AND PRINTERS.

1852.

